# *QuickStats:* Percentage* of Adults Aged ≥18 Years Who Were in Families Having Problems Paying Medical Bills in the Past 12 Months,^†^ by Disability Status^§^ and Age Group — United States, 2023

**DOI:** 10.15585/mmwr.mm7343a6

**Published:** 2024-10-31

**Figure:**
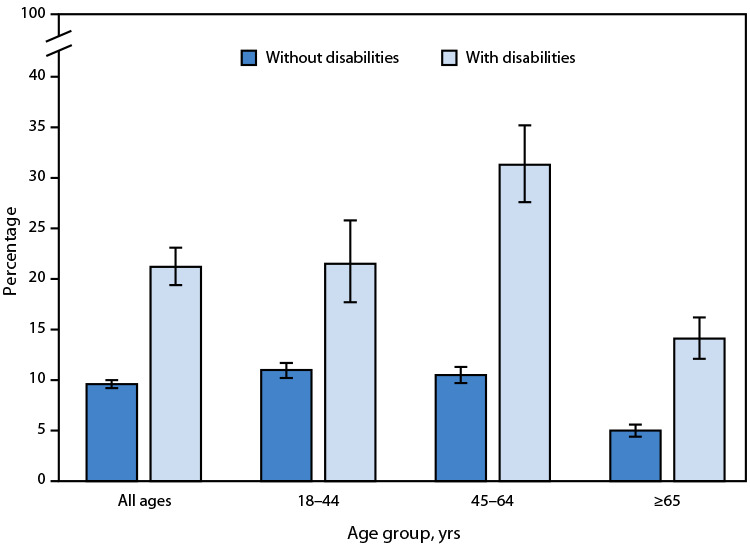
In 2023, the percentage of adults aged ≥18 years who were in families having problems paying medical bills in the past 12 months was higher among those with disabilities (21.2%) compared with those without disabilities (9.6%). This pattern was observed across all age groups.

**Supplementary Table**: https://stacks.cdc.gov/view/cdc/164154

**Source**: National Center for Health Statistics, National Health Interview Survey, 2023. https://www.cdc.gov/nchs/nhis.htm

